# Admission Cardiotocography as a Predictor of Low Apgar Score: An Observational, Cross-Sectional Study

**DOI:** 10.7759/cureus.14530

**Published:** 2021-04-17

**Authors:** Laila Nazir, Gul Lakhta, Khunsa Anees, Fahad R Khan, Sabah Safdar, Gul R Nazir, Mehwish I Irum, Safi U Khattak, Azra Salim

**Affiliations:** 1 Obstetrics and Gynaecology, Khyber Teaching Hospital, Peshawar, PAK; 2 Obstetrics and Gynaecology, Lady Reading Hospital, Peshawar, PAK; 3 Paediatrics, Services Institute of Medical Sciences, Lahore, PAK; 4 Cardiology, Lady Reading Hospital, Peshawar, PAK; 5 Radiology, Khyber Medical University, Peshawar, PAK; 6 Radiology, Khyber Teaching Hospital, Peshawar, PAK

**Keywords:** cardiotocography, ctg, low apgar score, neonatal mortality, electronic fetal monitoring, maternal and fetal medicine, fetal distress

## Abstract

Background and objective

Cardiotocography (CTG) has been used more frequently in recent decades to reduce intrapartum fetal mortality rates. The purpose of this study was to determine whether pathological or non-reactive CTG could predict a low Apgar (Appearance, Pulse, Grimace, Activity, and Respiration) score. An abnormal trace would indicate a distressed fetus, whereas a normal trace would indicate a well-oxygenated fetus.

Methods

This study included a total of 470 women with a gestational period of more than 37 weeks. Based on the results of their CTGs, they were divided into three groups. An emergency cesarean section (CS) was performed if there was any sign of fetal distress on CTG. The Apgar scoring for newborns was recorded in the proforma following delivery.

Results

The study was carried out at two major tertiary-care hospitals in Pakistan. A reactive CTG was found in more than one-third (39.36%) of the 470 patients. An Apgar score above 8 was obtained by 34.26% of the newborns, while an Apgar score below 8 was obtained by more than half (63.40%). Only 2.34% of newborns had an Apgar score below 6. A third (30.64%) of the patients had grade-1 meconium-stained liquor (MSL), 24.89% had grade-2 MSL, 19.79% had grade-3 MSL, and 24.68% had no MSL. One-third (32.34%) of the neonates were admitted to the neonatal intensive care unit (NICU) shortly after birth. When CTG was pathological or non-reactive, the odds of securing a higher Apgar score decreased by 70.45% (OR: 0.30; 95% CI: 0.20-0.44; p<0.001).

Conclusion

The main conclusion drawn from this study's findings is that a pathological CTG is an indicator of a low Apgar score.

## Introduction

The surveillance of fetal heart rate is a major element of antenatal care. The purpose of prenatal screening during childbirth is to recognize fetuses at risk of hypoxia during delivery and intervene if required to avoid fetal compromise [1-3]. Prior to the actual invention of the Pinard stethoscope, it was impossible to determine the fetus’s condition before birth. The only available option was to rely on the development of the uterus and the mother’s awareness of fetal activity. Eventually, the auscultation of fetal heart sounds and the condition of amniotic fluid were used to determine the fetus’s state during childbirth. Similarly, meconium-stained liquor (MSL) had been considered a typical sign of fetal distress.

Since its advent, cardiotocography (CTG) or intrapartum electronic fetal monitoring (EFM) has become a modern functional tool for fetal monitoring during pregnancy and delivery [4]. It measures fetal heart rate pattern and its relationship with uterine contractions [3]. CTG provides details regarding the fetal state. Its usage has gradually expanded over the past couple of decades and has contributed significantly to decreasing intrapartum fetal death rates [2]. With the emergence of modern technologies of EFM, there is a likelihood of lowering neonatal deaths due to hypoxia by 60% [2]. A normal trace is a sign of a well-oxygenated fetus, but an abnormal trace implies a distressed fetus [2]. It is important to note that CTG recording can neither act as a substitute for clinical expertise nor an excuse to leave the patient unsupervised during childbirth. Cesarean and instrumental deliveries have been on the rise over the last four decades [5]. CTG is a major contributor to the rise of operational deliveries. Yet, studies have reported incidences of false-positive CTG traces that had caused unnecessary operational deliveries [6]. CTG has a positive predictive value of 60% for fetal distress [6]. Besides its role in the rise in operational deliveries, CTG or EFM is considered superior to intermittent auscultation [7]. However, according to Beard et al., CTG is a subjective test; therefore, it alone should not be relied upon to avoid unnecessary interventions [8].

The regular use of admission CTG in low-risk pregnancies has been linked to a rise in operational deliveries with no decline in perinatal mortality [9]. Some health professionals have proposed that CTG should be performed only in cases with a strong probability of potential fetal hypoxia/acidosis related to maternal, medical, or fetal problems. This issue of risk versus benefits related to admission CTG has created a gap in the literature. Our primary objective in carrying out this study was to fill this gap, keeping in view the limitations of CTG and to prove the authenticity of admission CTG and its role in predicting fetal distress [taking low Apgar (Appearance, Pulse, Grimace, Activity, and Respiration) scores as a measure of fetal distress]. This study aimed to evaluate the role of pathological or non-reactive CTG in predicting low Apgar scores.

## Materials and methods

We conducted a cross-sectional, observational study in the Khyber Teaching Hospital (KTH) and Lady Reading Hospital (LRH) in Peshawar, Pakistan from April 1, 2020, to September 30, 2020. These two hospitals are the main tertiary-care centers in the Khyber Pakhtunkhwa state of Pakistan. The study enrolled 470 patients with a gestational period of over 37 weeks. Non-probability consecutive random sampling was used as the sampling method.

The approval from the Ethical Review Committees and informed consent from patients were obtained prior to the commencement of the study. Admission CTGs were carried out in the participants. Patients were categorized into three groups based on the results of their CTGs: (1) patients with reactive CTGs, (2) patients with non-reactive CTGs, and (3) patients with suspicious CTGs, as per the guidelines issued by the National Institute of Clinical Excellence (NICE) [10]. These CTGs varied based on the heart rate pattern and the absence or presence of acceleration, variability, and deceleration. MSL was recorded and graded accordingly. Grade-1 MSL was translucent and light yellow-green in color, grade-2 MSL was opalescent with deep green and light yellow in color, and grade-3 was opaque and deep green in color.

Upon any sign of fetal distress on CTG (i.e., baseline fetal heart rate below 110 or above 160 along with loss of variability or late decelerations), an emergency cesarean section (CS) was performed. After delivery, the newborn’s Apgar score was recorded at five minutes in the patient’s proforma along with other demographic details.

Inclusion criteria

We only enrolled those patients who met the eligibility criteria, which were as follows: gestational ages ranging from 37 to 42 weeks, ages ranging from 18 to 40 years, and both primigravida and multigravida.

Exclusion criteria

Patients with multiple pregnancies, known fetal anomalies, gestational diabetes, and pre-eclampsia were excluded to reduce the confounding effect. Fetuses with Cord-Around-the Neck (CAN; diagnosed on ultrasonography) were excluded as well.

Data analysis

Data were analyzed using SPSS Statistics version 25.0 (IBM, Armonk, NY). We presented continuous variables as mean ± standard deviation, while categorical variables were presented as frequencies and percentages. An independent t-test was conducted to assess if there were significant differences in age and BMI between the CTG results. A chi-square test was carried out for categorical variables, taking a p-value of ≤0.05 as significant. After controlling for age and BMI, we used an ordinal logistic regression analysis to see if differences in CTG results could explain the odds of newborns having different Apgar scores.

## Results

This study was conducted at the Obstetrics and Gynecology departments of two tertiary-care hospitals in Pakistan, with a view to determining the frequency of low Apgar scores in pregnant women with abnormal CTG findings. The age range of the patients was 23-40 years, and the mean patient age was 30.19 years (SD=5.73, Mdn=30.00); 203 patients (43.19%) were between 15-30 years, while 267 (56.81%) were between 31-45 years of age. Our sample’s BMI was in the range of 20.75-35 kg/m^2^, and the mean BMI was 27.04 (SD=5.28, Mdn=23.18). Of the total 470 patients, 185 (39.36%) had a normal or reactive CTG, 28 (5.96%) patients had a suspicious CTG, while 257 (54.68%) patients exhibited a pathological or non-reactive CTG. Among the 470 patients, 225 (47.77%) had a cesarean delivery, while 246 (52.23%) had a normal vaginal delivery. The Apgar score at five minutes of delivery was calculated; 161 (34.26%) of the newborns had an Apgar score above 8, 298 (63.40%) had an Apgar score below 8, and 11 (2.34%) of the newborns had an Apgar score below 6. Among the total participants, grade-1 MSL was found in 144 (30.64%) patients; 117 (24.89%) had grade-2 MSL, 93 (19.79%) had grade-3 MSL, and no MSL was noted in 116 (24.68%) patients. Upon delivery, 152 (32.34%) of the newborns were admitted to the neonatal Intensive care unit (NICU). These results are presented in Table 1.

**Table 1:** Summary of all the categorical and continuous variables used in the study SD: standard deviation; Mdn: median; Apgar: Appearance, Pulse, Grimace, Activity, and Respiration

Variable	Mean	SD	Mdn	N	%
Age, years	30.19	5.73	30		
Body mass index (BMI), kg/m^2^	27.04	5.28	23.18		
Age group					
15-30 years				203	43.19
31-40 years				267	56.81
Cardiotocography (CTG)					
Reactive/normal				185	39.36
Suspicious				28	5.96
Pathological/non-reactive				257	54.68
Apgar score					
>8				161	34.26
<8				298	63.4
<6				11	2.34
Meconium-stained liquor (MSL)					
None				116	24.68
Grade-1				144	30.64
Grade-2				117	24.89
Grade-3				93	19.79
Neonatal intensive care unit (NICU)					
Yes				152	32.34
No				318	67.66

We then compared continuous and categorical variables among the subgroups of CTG using t-test and chi-square test, as shown in Table 2 and Table 3.

**Table 2:** Comparison of the continuous variables among the subgroups of CTG using t-test SD: standard deviation; Mdn: median; CTG: cardiotocography

Variable	Mean	SD	N	Mdn	P-value
Age, years					0.003
Reactive/normal	29.097	5.771	185	26.000	
Suspicious	30.071	5.843	28	30.500	
Pathological/non-reactive	30.996	5.572	257	32.000	
Body mass index, kg/m^2^					0.071
Reactive/normal	26.928	5.308	185	23.183	
Suspicious	24.967	5.237	28	22.161	
Pathological/non-reactive	27.352	5.239	257	23.183	

**Table 3:** Comparison of the categorical variables among the subgroups of CTG using chi-square test Apgar: Appearance, Pulse, Grimace, Activity, and Respiration; CTG: cardiotocography

Variable	Reactive/normal, n (%)	Suspicious, n (%)	Pathological/non-reactive, n (%)	P-value
Age group				<0.001
15-30 years	100 (54%)	13 (46%)	90 (35%)	
31-40 years	85 (46%)	15 (54%)	167 (65%)	
Apgar score				<0.001
<8	85 (46%)	15 (54%)	198 (77%)	
>8	98 (53%)	11 (39%)	52 (20%)	
<6	2 (1%)	2 (7%)	7 (3%)	
Meconium-stained liquor (MSL)				<0.001
None	91 (49%)	10 (36%)	15 (6%)	
Grade-1	81 (44%)	9 (32%)	54 (21%)	
Grade-2	13 (7%)	5 (18%)	99 (39%)	
Grade-3	0 (0%)	4 (14%)	89 (35%)	
Cesarean section (CS)				<0.001
Yes	5 (3%)	9 (32%)	226 (88%)	
No	180 (97%)	19 (68%)	32 (12%)	
Neonatal intensive care unit (NICU)				<0.001
Yes	0 (0%)	0 (0%)	152 (59%)	
No	185 (100%)	28 (100%)	105 (41%)	

The regression coefficient for age, BMI, and suspicious CTG were not significant, suggesting that they did not affect the Apgar score. The regression coefficient for non-reactive CTG was significant, indicating that the odds of observing a higher Apgar score decreased by 70.45% when CTG was pathological or non-reactive (OR: 0.30; 95% CI: 0.20-0.44; p<0.001). The results of the ordinal logistic regression analysis are shown in Table 4.

**Table 4:** Ordinal logistic regression results for age, BMI, and CTG as predictors of Apgar score B: unstandardized beta; SE: standard error; OR: odds ratio; χ^2^: chi-square test statistic; CI: confidence interval; BMI: body mass index; CTG: cardiotocography; Apgar: Appearance, Pulse, Grimace, Activity, and Respiration

Predictor	B	SE	χ^2^	P-value	OR	95% CI
Age	-0.01	0.02	0.2	0.657	0.99	0.96-1.03
Body mass index (BMI)	0	0.02	0.03	0.87	1	0.97-1.04
Suspicious CTG	-0.13	0.4	0.11	0.741	0.88	0.40-1.92
Pathological/non-reactive CTG	-1.22	0.21	34.19	<0.001	0.3	0.20-0.44

## Discussion

In this study, we analyzed the link between admission CTG during labor and its implications on perinatal morbidity and mortality. EFM has been instrumental in decreasing perinatal mortality and illness. We discovered that pathological CTG is associated with a low Apgar score and NICU admissions. The mean age in our study group was 30.21 ± 5.73 years, while most patients were in the age group of 31-45 years. Though most of our patients were not overweight, our sample’s mean BMI was 27 ± 5.28 kg/m^2^. Of the total 470 patients, more than a quarter had a normal or reactive CTG; a small proportion had a suspicious CTG, while more than half of the patients exhibited a pathological or non-reactive CTG. Apgar score at five minutes of delivery was calculated. A third of the neonates had an Apgar score above 8, two-third had an Apgar score below 8 but above 6, and only 2% had an Apgar score below 6.

Chaudhari et al. reported reactive CTGs in 74% of their study sample, suspicious CTGs in 16%, and pathological traces in 10% [9]. On the contrary, in our study, 39% had reactive CTG, 6% had suspicious CTG, and 55% had a pathological CTG. In their study on 200 low-risk patients, Hegde et al. reported that fetal distress was linked to suspected and pathological CTG on admission. Therefore, the frequency of operational deliveries also continued to increase [11]. These findings were consistent with our findings.

Das et al. carried out a study to determine the usefulness of the CTG at admission in forecasting the risk of fetal distress during labor. The frequency of fetal distress and operational delivery was relatively high in the non-reassuring CTG group [12]. In our study, none of the patients with reassuring CTGs had their neonates admitted to NICU. Only two patients out of 470 had babies with an Apgar score below 6, despite a normal CTG trace. These findings favor the authenticity of admission CTG.

Furthermore, a study done by Rahman et al. reached findings similar to our study. Out of the total patients with reactive CTG, almost 10% of patients had MSL, and 6.5% were admitted to NICU. MSL and NICU admissions were higher by nearly fourfold in the suspicious group compared to patients with reactive CTG. Simultaneously, there was a tenfold increase in MSL and low Apgar score in the pathological group [13]. In our study, 7% of patients with reactive CTG had grade-2 MSL, which increased to 18% in patients with suspicious traces and 39% in patients with pathological traces.

According to a study by Gupta et al., CTG is not a good index of fetal distress; despite reassuring CTG, 34.6% of infants were born with MSL, one-third had an Apgar score below 7, and around 20% were admitted to the NICU [14]. Our study contradicted Gupta et al.’s findings because all of our patients admitted to NICU had a pathological CTG on admission. Moreover, our study endorsed findings by Panda et al. In their study, patients with reassuring CTG had reduced MSL rates (4.65%); only 3.48% had an Apgar score below 7, and 9.3% had NICU admissions. While patients in whom CTG was not reassuring, MSL increased dramatically to 85.71%, one-third had a low Apgar score, and 78.57% were admitted to NICU [15]. In our study, the percentages of grade-2 MSL, Apgar scores below 8, and NICU admission in cases with reactive CTGs were 7%, 46%, and 0% compared to 39%, 77%, and 59% in non-reactive CTG.

Mires et al. reported false-positive traces in 21.8% of patients who underwent operational delivery; among them, only 3.6% were non-reactive [6]. While in our sample, 20% had a non-reactive CTG, but the Apgar score above 8. Impey et al. demonstrated one-third of the traces to be non-reactive [16]. While in our study, more than half of the CTG traces were non-reactive.

In the study by Kansal et al., fetal distress was present in 16% of patients with normal admission CTG, 63% with suspicious CTG, and 97% with pathological CTG [17]. In comparison, fetal distress was seen in 46%, 54%, and 77% of our sample’s normal, suspicious, and pathological CTGs respectively. Bix et al. analyzed three trials and found that the admission CTG was not effective in predicting neonatal outcomes [18]. However, our study contradicts the statement of Blix et al. because our research concluded that a pathological CTG trace at admission could be a predictor of a low Apgar score (OR: 0.30; 95% CI: 0.20-0.44; p<0.001).

Multiple studies have endorsed that CTG plays a decisive role in detecting fetal distress and works as a harbinger of poor neonatal outcomes. A few among those studies are as follows: a study by Akhavan et al. conducted on 425 patients, a study by Sunitha et al. conducted on 100 patients, and the study by Kumar et al. conducted on 320 patients in 2019 [19-22]. Our study also backed the findings of these studies.

Gourounti et al. carried out a meta-analysis of multiple randomized controlled trials (RCTs) and determined that the admission CTG was connected to a rise in operational deliveries [22]. In contrast, in our study, only three patients with reactive CTG traces and six patients with suspicious traces underwent a CS. Larger randomized controlled trials are required to provide more insight into the use of CTG to predict neonatal outcomes and reduce perinatal mortality.

Limitations

Our study has several limitations. Firstly, we did not focus on the primary cause of a suspicious or pathological CTG, its reversibility, or the delivery timing to avoid excessive fetal hypoxia/acidosis and unnecessary obstetric intervention. Secondly, no additional testing was performed to evaluate fetal oxygenation. Moreover, our study was limited to two hospitals; hence, larger randomized trials are needed to back our findings in this part of the world.

## Conclusions

The CTG is an easy, painless, and cost-effective test for evaluating fetal well-being. It could be used as an effective screening investigation. This test can identify patients who are at high risk for operational delivery. Our research focused on the importance of CTG in detecting fetal distress. The study’s prediction of a low Apgar score in cases of a non-reactive CTG trace illustrated the significance of CTG in detecting fetal distress. Our study’s findings can prove instrumental in decreasing the need for continuous CTG monitoring or other invasive testing required to detect fetal distress, thereby saving time and resources and mitigating patient discomfort, particularly in those hospitals with a high flow of patients.
